# Treatment with CFTR Modulators for Cystic Fibrosis: What a Pediatric Gastroenterologist Needs to Know

**DOI:** 10.3390/children12091104

**Published:** 2025-08-22

**Authors:** David Gonzalez Jimenez, Ruth García Romero, Alejandro Rodríguez Martínez, Saioa Vicente Santamaria

**Affiliations:** 1 Cystic Fibrosis Unit, Hospital Universitario Central de Asturias, 33011 Oviedo, Spain; 2 Hospital Infantil Miguel Servet, 50009 Zaragoza, Spain; ruthgarciaromero@yahoo.es; 3 Pediatric Gastroenterology, UGC de Pediatría Hospital Infantil—HH.UU, Virgen del Rocío Avda, Manuel Siurot s/n, 41013 Sevilla, Spain; alejandro.rodriguez.m.sspa@juntadeandalucia.es; 4 Cystic Fibrosis Unit, Hospital Universitario Ramon y Cajal, Ctra, de Colmenar Viejo km. 9,100, 28034 Madrid, Spain; saioa.vicente@salud.madrid.org

**Keywords:** cystic fibrosis, CFTR modulators, ETI, nutritional assessment, cystic fibrosis-related bone disease, CFBD, cystic fibrosis liver disease, CFLD

## Abstract

**Highlights:**

**What are the main findings?**

- mCFTRs, particularly ETI therapy, significantly improve gastrointestinal function and nutritional status and reduce intestinal inflammation in pediatric patients with cystic fibrosis. These improvements are accompanied by enhanced levels of fat-soluble vitamins and changes in body composition.
- By restoring CFTR protein function, modulator therapy may positively influence bone density and structure through both direct effects on bone cells and indirect mechanisms related to improved overall health.
- Pediatric gastroenterologists must incorporate modulator-driven changes into routine clinical monitoring, with special attention to nutrition, bone density, and hepatobiliary markers.
- These findings support a shift toward personalized, multidisciplinary care strategies that reflect the expanded systemic benefits and emerging risks of mCFTRs in children.

**Abstract:**

**Background:** Cystic fibrosis (CF) is a multisystemic disorder caused by CFTR gene mutations, leading to impaired protein function and affecting pulmonary, gastrointestinal, hepatobiliary, skeletal, and nutritional health. The advent of CFTR modulators—especially the triple therapy elexacaftor/tezacaftor/ivacaftor (ETI)—has revolutionized clinical management, offering genotype-specific benefits beyond pulmonary outcomes. Pediatric gastroenterologists must now recognize and address emerging gastrointestinal and nutritional challenges introduced by modulator therapy. **Methods**: A narrative review was conducted to assess the impact of CFTR modulators on gastrointestinal function, nutritional status, bone health, and hepatobiliary involvement in pediatric patients. A structured literature search was performed using PubMed, EMBASE, and Scopus databases. Filters included articles in English or Spanish. Following full-text review based on relevance and quality, 68 articles were selected for inclusion in this review. **Results**: CFTR modulators have demonstrated potential improvements in gastrointestinal function, nutrient absorption, weight gain, and bone mineral density. In pediatric populations, ETI therapy has been associated with early increases in lean mass, enhanced vitamin levels, and promising trends in bone microarchitecture. However, variable outcomes regarding liver function and bone mineral density highlight the need for careful monitoring. **Conclusions**: While CFTR modulators present novel opportunities for systemic improvement in CF, their long-term impact on digestive and skeletal health in children remains under investigation. Pediatric gastroenterologists play a pivotal role in monitoring nutritional and hepatobiliary outcomes, optimizing treatment plans, and guiding personalized care strategies in the era of CFTR modulation.

## 1. Introduction

Cystic fibrosis (CF) is a multisystemic autosomal recessive disorder caused by mutations in the cystic fibrosis transmembrane conductance regulator (CFTR) gene, resulting in dysfunctional CFTR protein expression and impaired epithelial chloride and bicarbonate transport. Although traditionally recognized for its pulmonary manifestations, CF has extensive extrapulmonary involvement, particularly affecting the gastrointestinal tract, liver, pancreas, bone metabolism, and overall nutritional status.

The discovery of the CFTR gene in 1989 marked a turning point in the understanding and treatment of CF. The realization that specific mutations in the CFTR gene lead to defective or absent protein function prompted a shift toward precision medicine. This led to the development of CFTR modulators (mCFTRs). Ivacaftor, approved in 2012, became the first drug to improve the activity of CFTR protein in patients with gating mutations. Subsequent combination therapies, including lumacaftor/ivacaftor and elexacaftor/tezacaftor/ivacaftor (ETI), have broadened treatment options to cover a larger proportion of the CF population.

mCFTRs work by improving the function of the defective CFTR protein at different stages of its cellular processing and activity. They are classified into categories based on their action: *correctors* (e.g., lumacaftor, tezacaftor, and elexacaftor) help the CFTR protein fold properly and reach the cell membrane, while *potentiators* (e.g., ivacaftor) enhance the channel’s ability to transport chloride ions across the membrane. Together, these modulators can significantly reduce disease burden, improve lung function, and enhance quality of life for individuals with CF.

The approval and widespread use of mCFTRs have significantly enhanced pulmonary function and survival. However, their systemic effects are now garnering increasing attention due to their impact on mucus rheology, gastrointestinal motility, nutrient absorption, intestinal inflammation, hepatic biomarkers, and bone mineralization.

Pediatric gastroenterologists are uniquely positioned to address both the therapeutic benefits and potential metabolic or organ-specific consequences of CFTR modulation. With the expansion of modulator eligibility to younger age groups—including infants and toddlers—there is a critical need to revise assessment protocols involving nutrition, hepatobiliary function, and musculoskeletal development. Recognizing the systemic implications of CFTR restoration is essential for delivering comprehensive, multidisciplinary care and optimizing long-term outcomes in this evolving therapeutic landscape.

## 2. Materials and Methods

This review aims to synthesize current evidence regarding the impact of mCFTRs on gastrointestinal function, nutritional status, bone health, and hepatobiliary involvement in pediatric patients, and to highlight the clinical considerations pediatric gastroenterologists must keep in mind when managing this population. This work was conducted as a narrative review, aiming to provide a comprehensive, clinically oriented overview based on existing evidence.

### Search Strategy

A systematic literature search was performed across three major biomedical databases, PubMed, Scopus, and EMBASE, covering articles published up to March 2025. The following Boolean search strategy was applied:

“Cystic Fibrosis”[Mesh] AND (“CFTR Modulators”[tiab] OR “ivacaftor”[tiab] OR “lumacaftor”[tiab] OR “tezacaftor”[tiab] OR “elexacaftor”[tiab] OR “CFTR protein, human”[Supplementary Concept]).

Filters were applied to include studies published in English or Spanish. Additionally, a date range filter was applied, restricting the search to publications released between 1 March 2005 and 1 March 2025. No restrictions were placed on study design. All selected articles were screened for relevance, methodological rigor, and alignment with the scope of this review.

After removing duplicates and screening titles and abstracts, a total of 2986 articles were initially identified. Following full-text review based on relevance and quality, 68 articles were selected for inclusion in this review.

## 3. Results

### 3.1. mCFTRs and Gastrointestinal Changes

#### 3.1.1. Gastrointestinal Dysfunction in the Clinical Course of CF

CF not only compromises pulmonary function but also significantly affects the gastrointestinal system due to dysfunction of the CFTR protein. This protein is actively expressed on the apical membrane of enterocytes, where it regulates the secretion of chloride and bicarbonate ions into the intestinal lumen. This function is essential for maintaining luminal fluidity and ensuring an appropriate intestinal pH. Bicarbonate, in particular, plays a key role in neutralizing acidic gastric contents, thus promoting an optimal environment for enzymatic activity and the proper digestion and absorption of essential nutrients. Additionally, bicarbonate contributes decisively to mucus solubilization and expansion. When its secretion is reduced, as occurs in CF, mucus becomes thicker and more adhesive, impairing intestinal transit and promoting luminal obstruction. These changes in mucus consistency favor the accumulation of undigested material and create a microenvironment conducive to uncontrolled bacterial proliferation, resulting in an imbalance of the gut microbiota or dysbiosis [1]. This dysbiosis, characterized by an increase in pathogenic bacteria and a decrease in beneficial species, may further exacerbate inflammation through sustained activation of the intestinal immune system. The chronic presence of an inflammatory environment and altered intestinal motility contribute to persistent gastrointestinal symptoms, including abdominal pain, distension, malabsorption, and abnormal bowel habits. Over time, these alterations may lead to a state of chronic intestinal inflammation, which is associated with an increased risk of complications and nutritional compromise [2].

Several studies have demonstrated that CFTR gene variants are associated with significant changes in the composition of the intestinal microbiota. In particular, patients who are homozygous for the F508del mutation—associated with a severe disease phenotype—exhibit a dysbiotic profile with increased relative abundance of potentially pathogenic bacterial species, such as Escherichia coli. At the same time, there is a marked reduction in beneficial species, notably Faecalibacterium prausnitzii, an anti-inflammatory microorganism that plays a key role in maintaining intestinal homeostasis [3].

#### 3.1.2. Impact of mCFTRs on Gastrointestinal Function in CF

Recent studies have also highlighted the impact of mCFTRs such as ivacaftor on the gut microbiota, associating treatment with decreased intestinal inflammation and increased abundance of beneficial bacteria such as Akkermansia [4].

Combination therapy with elexacaftor, tezacaftor, and ivacaftor (ETI) has shown significant improvements in gastrointestinal outcomes—a domain that has traditionally posed considerable morbidity in people with CF (PwCF). One of the most relevant instruments to assess the burden of digestive symptoms in these patients is the CFAbd-Score, a validated tool specifically designed to quantify CF-related gastrointestinal symptoms. Several studies have reported significant improvement in the CFAbd-Score following 24 weeks of ETI treatment, reflecting reductions in the frequency and severity of symptoms such as abdominal pain, gastroesophageal reflux, altered bowel habits (constipation or diarrhea), and appetite loss. Furthermore, patients report subjective improvements in their digestive health-related quality of life, which positively impacts treatment adherence and overall well-being. These benefits may be attributed to enhanced CFTR function in the intestinal epithelium, resulting in better mucus hydration, improved gastrointestinal motility, and a more favorable environment for nutrient absorption and digestion [5].

mCFTRs have also been associated with significant reductions in fecal inflammatory markers such as pyruvate kinase M2 and calprotectin, supporting the improvements observed in gastrointestinal symptoms and suggesting effective mitigation of intestinal inflammation [6]. A recent Spanish multicenter study confirmed the positive effects of these therapies on intestinal inflammation as measured by fecal calprotectin. The study, which included 117 pediatric and adolescent patients, compared dual therapies (lumacaftor/ivacaftor or tezacaftor/ivacaftor) with triple therapy involving ETI. Results demonstrated a significant reduction in intestinal inflammation in both groups, with a more pronounced effect in those receiving ETI [7].

mCFTRs show great promise in improving mucosal hydration and potentially altering both pulmonary and gastrointestinal microbiota, emphasizing the importance of the gut–lung axis in CF pathophysiology. mCFTRs may thus contribute not only to respiratory improvement but also to partial restoration of gastrointestinal homeostasis, with potential implications for systemic inflammatory responses and patient quality of life [8].

#### 3.1.3. Gastrointestinal Side Effects in PwCF Undergoing mCFTRs

Although mCFTRs have markedly enhanced gastrointestinal function in pediatric patients with CF, gastrointestinal adverse events remain relatively frequent during therapy, affecting approximately 15% of treated individuals. These include abdominal discomfort, flatulence, nausea, and alterations in bowel habits, such as diarrhea or constipation. In most cases, symptoms are mild and manageable with symptomatic treatment or dose adjustment and rarely require discontinuation of therapy.

A more serious but infrequent complication is acute pancreatitis, which has been reported predominantly in patients who experience partial recovery of pancreatic exocrine function following mCFTRs, although cases have also been observed in individuals who remain pancreatic insufficient. A recent pediatric case series documented a median onset of 30 months after treatment initiation. These findings underscore the importance of close monitoring during treatment initiation and ongoing follow-up to distinguish drug-related effects from underlying disease manifestations.

### 3.2. Nutritional Assessment and Support in PwCF

#### 3.2.1. Nutritional Challenges in PwCF

CF is a multisystem genetic disorder that significantly affects nutritional status, particularly in pediatric patients. Malabsorption due to pancreatic insufficiency, increased energy expenditure from chronic respiratory infections, and gastrointestinal complications contribute to growth failure and poor nutritional outcomes. Adequate nutrition is essential not only for supporting growth and development but also for optimizing pulmonary function and overall clinical prognosis. Early and aggressive nutritional intervention has been shown to improve survival rates and quality of life in children with CF.

Evaluating nutritional status in children with CF requires a comprehensive and multidisciplinary approach. Standard assessments include anthropometric measurements such as weight, height, and body mass index (BMI), alongside growth velocity and percentiles. Biochemical markers, including serum levels of fat-soluble vitamins (A, D, E, and K), albumin, and prealbumin, provide insight into micronutrient deficiencies and protein status. Additionally, body composition analysis using tools like bioelectrical impedance (BIA) can help detect changes in lean mass and fat stores. Regular monitoring is crucial to guide individualized nutritional strategies and ensure optimal health outcomes.

#### 3.2.2. Nutritional Assessment and Support in PwCF Undergoing mCFTRs

Treatment with ETI has been associated with an increase in body weight and, consequently, in BMI across all age groups, leading to a reduction in the prevalence of undernutrition and a rise in overweight and obesity [9]. When such weight gain reflects an increase in lean body mass, it may result in improved pulmonary function; however, excessive accumulation of fat mass is linked to poorer cardiovascular and metabolic health [10,11]. The mechanisms underlying this weight gain are believed to involve enhanced intestinal absorption—driven by increased intestinal pH and reduced inflammation—along with decreased basal energy expenditure due to fewer pulmonary exacerbations and improved pancreatic exocrine function, particularly in patients who initiated ETI early. Contrary to initial assumptions, studies have not demonstrated an increase in caloric intake; on the contrary, a reduction has been observed in certain age groups. Additionally, a substantial proportion of patients experience weight gain within the first months of treatment, underscoring the importance of conducting a thorough nutritional assessment prior to ETI initiation to identify those at higher nutritional risk (especially overweight or obese individuals), and ensuring close follow-up during the early stages of therapy.

In nutritional assessment, reliance solely on anthropometric parameters such as weight and height is insufficient. The systematic use of body composition techniques—or, at a minimum, anthropometric measurements such as mid-upper arm circumference and skinfold thickness—should be encouraged when more advanced tools are unavailable [12]. The expanded use of morphofunctional assessments, including BIA, ultrasound, and handgrip dynamometry in patients over six years of age, can support clinical decisions regarding dietary interventions and physical activity. The overarching goal is to ensure that weight gain following ETI initiation translates into increases in lean and cellular mass, as well as improvements in muscle quality, as evaluated through ultrasound and dynamometry.

One parameter gaining particular relevance in morphofunctional assessments is the phase angle. In adults, values below 4 have been associated with increased morbidity and mortality in conditions such as chronic kidney disease, HIV, and cancer; however, no validated cutoff points have yet been established for CF. In children, phase angle values vary according to age and sex—being higher in males and increasing progressively until the age of 18—which further emphasizes the need for age- and population-specific reference standards [13]. Most importantly, periodic assessments of phase angle are recommended to track individual patient evolution based on prior measurements.

Unlike adult patients, in whom weight gain predominantly reflects increased fat mass, pediatric patients appear to exhibit a more balanced gain in both fat and lean compartments, with notable interindividual variability [14,15,16]. Nonetheless, current studies are still too preliminary to draw definitive conclusions. These findings may be related to the dynamic developmental stage of children, the shorter duration of disease progression, and the close monitoring provided by multidisciplinary care teams from an early age, especially following the implementation of universal newborn screening programs.

Collectively, these observations indicate that, in ETI-treated patients, nutritional excess disorders have now surpassed deficiencies, both in the short and long term. The conventional recommendation of prescribing hypercaloric diets (ranging from 120–200% of the RDAs) is now considered outdated and should be individualized. As such, recent guidelines on nutritional support advocate for tailored dietary recommendations, with a stronger emphasis on healthy eating patterns and nutrient quality (e.g., fruits, vegetables, legumes, and whole grains) over caloric quantity [17]. Nutritional quality of calorie-rich but not nutrient-dense foods traditionally consumed by CF patients may have long-term implications on cardiovascular and metabolic health as people with CF live longer. The role of exercise for cardiovascular health and in maintaining a healthy weight also needs to be emphasized in these patients.

Studies on fat-soluble vitamin supplementation have produced variable findings; however, ETI treatment generally improves serum levels of vitamin A and, to a lesser extent, vitamins D and E [18]. In addition to improved intestinal absorption and pancreatic function, the reduction in systemic inflammation may contribute to elevated retinol concentrations, as retinol is an acute-phase reactant. A few symptomatic cases of hypervitaminosis A—manifesting as benign intracranial hypertension—have been reported, although most presented only modest elevations in serum levels [19]. Consequently, close monitoring of vitamin concentrations following ETI initiation is recommended to ensure appropriate dose adjustments in supplementation.

### 3.3. Cystic Fibrosis-Related Bone Disease (CFBD)

Cystic fibrosis-related bone disease (CFBD) is a well-recognized complication in patients with CF, characterized by low bone mineral density (BMD) and an elevated risk of fractures [20,21,22,23,24,25,26]. Bone loss begins early in life and worsens with increasing age. The prevalence of osteoporosis in adults has been estimated at 23.5%, and that of osteopenia ranges between 35–45% [27,28]. With the progressive increase in life expectancy among individuals with CF, CFBD has become more prevalent and clinically significant.

The most evident symptom of CFBD is the increased frequency of fractures, particularly in the ribs and vertebrae [29]. These may result in significant pain, deformities, and a reduction in lung volume, cough efficacy, and airway clearance—ultimately leading to more frequent pulmonary exacerbations and faster decline in lung function.

#### 3.3.1. Management of CFBD

Non-pharmacological and general management includes improvement of nutritional status, ensuring adequate intake of calcium, vitamin D, and vitamin K [30,31], and appropriate use of pancreatic enzyme replacement therapy; optimization of pulmonary disease management and infection control to minimize exacerbations; encouragement of weight-bearing physical activity; avoidance or minimization of medications known to impair bone health, such as glucocorticoids or proton pump inhibitors (PPIs); addressing secondary causes of low BMD such as hypogonadism or CFRD [30]; and assessment of BMD through dual-energy X-ray absorptiometry (DXA), which is the standard recommended tool for screening, diagnosis, and follow-up [20,29].

Pharmacological treatment: Bisphosphonates are considered first-line treatment for clinically significant bone disease and in transplant candidates [24,25,26,30]. These are available in oral forms (alendronate, risedronate, and ibandronate) and intravenous forms (ibandronate, pamidronate, and zoledronic acid) and have shown to improve BMD in both adults and children. However, their use in pediatric populations remains controversial due to long-term safety concerns [30]. Moreover, evidence regarding fracture reduction in CF patients is limited [32].

Emerging agents include denosumab (a monoclonal antibody that inhibits RANKL), anabolic agents such as teriparatide (a PTH analog) and abaloparatide [33], and selective estrogen receptor modulators (SERMs) like raloxifene and bazedoxifene. These are possible therapeutic options, but further studies are needed in CF populations.

#### 3.3.2. mCFTRs and CFBD

mCFTRs are a class of drugs developed to target the underlying genetic defect in CF: CFTR protein dysfunction [34]. While their main therapeutic effect is the improvement of pulmonary function and nutritional status, emerging evidence suggests a potential impact on CFBD as well [35,36]. Research into their effects on BMD and bone structure in CF patients is still in early stages, but initial findings are encouraging.

##### Direct Effects on Bone Cells

In vitro studies have shown that mCFTRs may improve certain intrinsic abnormalities in bone cells. For example, a study involving samples from four subjects with the F508del mutation demonstrated that treatment with C18, a CFTR corrector, significantly reduced RANKL protein production in stimulated human osteoblasts [37]. Moreover, modulators have been shown to improve the altered RANKL/OPG ratio and reduce PGE2 production in F508del-mutant osteoblasts. In murine models carrying the F508del mutation, activation of CFTR channels with correctors such as miglustat led to improvements in bone formation, mass, and microarchitecture. These findings suggest that mCFTRs may exert a direct effect on bone remodeling.

##### Clinical Effects in Humans

The effects of mCFTRs on bone health in humans remain unclear, with studies reporting mixed findings. A small retrospective study involving seven adults treated with ivacaftor showed a significant improvement in lumbar spine BMD. A larger prospective study with 26 adults and children found no significant changes in BMD but did report notable improvements in cortical bone microarchitecture (volume, area, and porosity) in adults after two years of treatment with ivacaftor—independent of pulmonary function and BMI.

For patients homozygous for F508del, a study using lumacaftor/ivacaftor suggested a potentially positive effect on bone mineral metabolism after one year of treatment, although the observed change in total spine T-score in the small sample (four patients) was not statistically significant [38]. Lastly, a pilot study involving nine patients treated with elexacaftor/tezacaftor/ivacaftor reported significant increases in BMD at the hip and spine after three months of therapy, in addition to expected improvements in weight, BMI, and body composition [39]. However, another retrospective study from the UK including 97 patients assessed before and after starting elexacaftor/tezacaftor/ivacaftor over an average follow-up of 2.1 years showed no significant changes in BMD, despite improvements in BMI [40].

##### Indirect Effects

In addition to potential direct cellular effects, mCFTRs may improve bone health indirectly by positively influencing several CFBD risk factors such as nutritional status, vitamin absorption (especially vitamins D and K), pulmonary function, systemic inflammation, and physical activity capacity.

In summary, mCFTRs, by improving CFTR protein function, may positively influence bone density and structure in PwCF. This effect may occur both through direct actions on bone cells and indirectly via overall improvements in patient health. Initial findings—though often based on small sample sizes—are encouraging, demonstrating enhancements in BMD and/or bone microarchitecture. However, further long-term studies with larger patient cohorts are needed to confirm these results and fully understand the magnitude and durability of these effects. Ongoing investigations aim to shed more light on the impact of modulator therapies on bone health.

### 3.4. Hepatobiliary Involvement and mCFTRs

#### 3.4.1. Cystic Fibrosis (CF)-Related Hepatobiliary Involvement:

The hepatobiliary manifestations included within the spectrum of CF-associated liver disease are extremely varied and can present with different levels of severity at various stages of life [41]. The most frequent alterations include neonatal cholestasis and elevated serum levels of liver enzymes such as AST, ALT and GGT. Other possible complications include hepatic steatosis, cholelithiasis, multinodular cirrhosis, biliary cirrhosis, and non-cirrhotic portal hypertension [41,42].

Progression to liver cirrhosis in these patients is critical, as it is usually associated with portal hypertension (PHT) and oesophageal varices (EV), representing one of the main clinical complications. EVs are a significant cause of morbidity and mortality in individuals with CF and liver disease due to the risk of gastrointestinal bleeding and the complexity of their medical and surgical management [43].

Therefore, early recognition and close monitoring of hepatobiliary manifestations in CF patients are essential to prevent the progression of liver disease and its more severe consequences.

#### 3.4.2. Epidemiology

Liver disease in PwCF usually manifests in the first decade of life and is estimated to affect approximately 30–40% of patients before the age of 12 years [44]. Among these cases, about 10% of children and adolescents with liver disease progress to cirrhosis before reaching adulthood [45], which significantly contributes to this pathology being the third leading cause of mortality in PwCF, accounting for approximately 2.5% of overall mortality in this population [46].

In the adult population with CF, the prevalence of liver disease varies widely, estimated at between 13% and 45% according to different series and diagnostic methods used [47,48,49,50]. However, these figures may be underestimated, since postmortem studies have revealed the presence of focal cirrhosis in up to 72% of the cases analyzed [51], suggesting that there is a subclinical or undiagnosed component of liver involvement more frequently than what is detected during life.

In the pediatric population, the available data are equally variable and usually reflect somewhat lower prevalences, ranging from 9.5% to 30% according to the different published series [52,53]. It should be noted that a recent study carried out by the Cystic Fibrosis and Pancreas working group of the Spanish Society of Pediatric Gastroenterology, Hepatology and Nutrition (SEGHNP) has shown that the most common phenotype in the pediatric population is liver involvement without cirrhosis, with a mean age of diagnosis of around 7 years [54].

These figures underline the importance of close surveillance and early diagnosis of liver disease in CF, both in pediatric and adult age, to try to prevent progression to more severe complications such as cirrhosis and PHT.

#### 3.4.3. Hepatobiliary Involvement and mCFTRs

The hepatobiliary impact of mCFTRs in PwCF is complex and, to date, is still not completely defined, due to the heterogeneity of studies and the relative novelty of these treatments.

A retrospective study published by Ramsey et al. showed that PwCF treated with mCFTRs had a lower incidence of liver cirrhosis compared to those who did not receive these drugs or who used only ursodeoxycholic acid (UDCA) as treatment [55].

More recently, data from pediatric cohorts have been published showing that treatment with ETI in this population reduces liver stiffness—assessed by techniques such as elastography—and improves analytical parameters of liver function after 12 months of treatment [56]. However, these findings have not been consistent throughout the literature, as other studies have not observed significant improvements in liver function or fibrosis markers after initiation of ETI [57,58], reflecting the need for further research to clarify these potential benefits.

In a recently published pediatric cohort of 11 patients with advanced liver disease and PHT, it was observed that treatment with ETI was well-tolerated, with no serious adverse effects requiring discontinuation [59]. However, it is important to point out that in these cases the dosage of ETI did not follow the standard regimen, but rather individualized guidelines were applied, accompanied by very close analytical monitoring, with controls every 1–2 months to monitor possible hepatic complications.

Conversely, clinical trials conducted with mCFTRs have described an increase in liver transaminases as an adverse effect in approximately 5–10% of patients, although in most cases this increase was transient and exceptionally led to definitive discontinuation of treatment [60,61,62,63]. In addition, some authors have warned of the possibility that the use of ETIs is associated with an increase in hepatic stiffness and alterations in bile acid metabolism in certain PwCF, which could have clinical implications that are not yet fully clarified [64].

Due to the inconsistency of the available evidence, the Cystic Fibrosis Foundation cannot, at this time, issue a firm recommendation for or against the use of mCFTRs in CF patients with advanced, non-decompensated liver disease [65]. However, there is consensus against their use in patients with decompensated liver disease, given the potential risk of clinical worsening.

In conclusion, although mCFTRs have consistently demonstrated a positive impact on respiratory and nutritional parameters and quality of life in PwCF, it has not yet been confirmed that these drugs achieve reversal of liver fibrosis or sustained improvement of liver function test results in the short term [66]. It is essential to bear in mind that mCFTRs can have both beneficial and adverse effects on the liver, which underscore the need to carefully individualize the indication of these treatments, always assessing the risk–benefit ratio in each case and maintaining close clinical and analytical surveillance in those patients with pre-existing liver disease [67,68].

## 4. Conclusions

The development of mCFTRs has transformed the therapeutic landscape of CF, enabling targeted correction of the underlying molecular defect. While their pulmonary benefits are well-established, increasing attention is being paid to their systemic effects—particularly on gastrointestinal, nutritional, hepatic, and skeletal health.

From the perspective of pediatric gastroenterologists, these advances offer new opportunities but also introduce important clinical challenges. Current evidence suggests that mCFTRs improve intestinal absorption, reduce inflammation, and promote a more balanced gut microbiota, contributing to enhanced digestive function. Positive effects on nutritional status and serum levels of fat-soluble vitamins have also been observed, although long-term implications for bone health remain uncertain.

Hepatobiliary involvement presents a more complex picture. Some studies report improvements in liver stiffness and biochemical markers following modulator therapy, while others show no significant changes. Transient elevations in liver transaminases and potential alterations in bile acid metabolism have also been documented, underscoring the need for close monitoring—especially in patients with pre-existing liver disease.

As modulator therapy becomes available to increasingly younger populations, including infants and toddlers, clinical monitoring protocols must be adapted accordingly. In this evolving therapeutic era, pediatric gastroenterologists play a central role in delivering comprehensive care. A multidisciplinary approach is essential to optimize outcomes, ensure early detection of complications, and tailor interventions to the individual needs of each patient.

Ongoing research is critical to clarify the long-term systemic effects of mCFTRs and to guide evidence-based clinical decision-making in the care of children with CF.

## Abbreviations

The following abbreviations are used in this manuscript:

CF	Cystic fibrosis
CFTR	Cystic fibrosis transmembrane conductance regulator
mCFTRs	CFTR modulators
ETI	Elexecatftor-tezacaftor-ivacaftor
PwCF	People with cystic fibrosis
BMI	Body mass index
CFBD	Cystic fibrosis-related bone disease
DXA	Dual-energy X-ray absorptiometry
PHT	Portal hypertension
EV	Oesophageal varices
BIA	Bioelectrical impedance analysis
BMD	Bone mineral density
PPIs	Proton pump inhibitors
UDCA	Ursodeoxycholic acid
